# HTLV-1 Tax mediated downregulation of miRNAs associated with chromatin remodeling factors in T cells with stably integrated viral promoter

**DOI:** 10.1186/1742-4690-11-S1-P103

**Published:** 2014-01-07

**Authors:** Saifur Rahman, Kevin Quann, Devanshi Pandya, Shruti Singh, Zafar K Khan, Pooja Jain

**Affiliations:** 1 Drexel Institute for Biotechnology and Virology Research, and the Department of Microbiology and Immunology, Drexel University College of Medicine, Doylestown, PA, USA

## 

RNA interference (RNAi) is a natural cellular mechanism to silence gene expression and is predominantly mediated by microRNAs (miRNAs) that target messenger RNA. Viruses can manipulate the cellular processes necessary for their replication by targeting the host RNAi machinery. This study explores the effect of human T-cell leukemia virus type 1 (HTLV-1) transactivating protein Tax on the RNAi pathway in the context of a chromosomally integrated viral long terminal repeat (LTR) using a CD4(+) T-cell line, Jurkat. Transcription factor profiling of the HTLV-1 LTR stably integrated T-cell clone transfected with Tax demonstrates increased activation of substrates and factors associated with chromatin remodeling complexes. Using a miRNA microarray and bioinformatics experimental approach, Tax was also shown to downregulate the expression of miRNAs associated with the translational regulation of factors required for chromatin remodeling. These observations were validated with selected miRNAs and an HTLV-1 infected T cells line, MT-2. miR-149 and miR-873 were found to be capable of directly targeting p300 and p/CAF, chromatin remodeling factors known to play critical role in HTLV-1 pathogenesis. Overall, these results are first in line establishing HTLV-1/Tax-miRNA-chromatin concept and open new avenues toward understanding retroviral latency and/or replication in a given cell type.

